# Prediction of VMAT delivery accuracy with textural features calculated from fluence maps

**DOI:** 10.1186/s13014-019-1441-7

**Published:** 2019-12-23

**Authors:** Jong Min Park, Jung-in Kim, So-Yeon Park

**Affiliations:** 1 Department of Radiation Oncology, Seoul National University Hospital, Seoul, Korea; 2 Institute of Radiation Medicine, Seoul National University Medical Research Center, Seoul, Korea; 3 Biomedical Research Institute, Seoul National University Hospital, Seoul, Korea; 4 Robotics Research Laboratory for Extreme Environments, Advanced Institute of Convergence Technology, Suwon, Korea; 5 Department of Radiation Oncology, Veterans Health Service Medical Center, Seoul, Korea

**Keywords:** Modulation degree; plan delivery accuracy, Pre-treatment quality assurance, Textural feature, Volumetric modulated arc therapy

## Abstract

**Background:**

Comprehensively textural feature performance test from volumetric modulated arc therapy (VMAT) fluences to predict plan delivery accuracy.

**Methods:**

A total of 240 VMAT plans for various treatment sites were analyzed, with Trilogy and TrueBeam STx systems. Fluence maps superposed fluences at each control point per plan. The textural features were the angular second moment (ASM), inverse difference moment (IDM), contrast, variance, correlation, and entropy, calculated from fluence maps using three displacement distances. Correlation analysis of textural feature performance as predictors of VMAT delivery accuracy used global gamma passing rates with MapCHECK2 and ArcCHECK dosimeters, and mechanical delivery errors calculated from machine log files.

**Results:**

Spearman’s rank correlation coefficients (*r*) of the ASM (*d* = 10) to the gamma passing rates with 1%/2 mm using the MapCHECK2 were 0.358 and 0.519, respectively (*p* <  0.001). For the ArcCHECK, they were 0.273 (*p* = 0.001) and 0.259 (*p* = 0.009), respectively. The *r*-values of the ASM (*d* = 10) to the Trilogy and TrueBeam STx MLC errors were − 0.843 and − 0.859, respectively (*p* <  0.001), and those to the MU delivery errors were − 0.482 and − 0.589, respectively (*p* <  0.001). The ASM (*d* = 10) showed better performance in predicting VMAT delivery accuracy.

**Conclusions:**

The ASM (*d* = 10) calculated from VMAT plan fluence maps were strongly correlated with global gamma passing rates and MLC delivery errors, and can predict VMAT delivery accuracy.

## Background

Volumetric modulated arc therapy (VMAT) can deliver conformal prescription doses to target volumes while minimizing doses to proximal organs at risk (OARs) by generating steep dose gradients between the target volumes and the OARs [1–7], which is enabled by its use of photon beam modulations [8]. The modulated photon beams of VMAT can be rapidly delivered to a patient by simultaneously modulating multi-leaf collimator (MLC) positions, gantry rotation speeds, and dose-rates during a single or multiple rotations of a gantry around a patient [8]. Although the photon beam modulations of VMAT can generate optimal dose distributions, excessive modulations can result in discrepancies between planned and delivered dose distributions, leading to undesired clinical results [9, 10]. Because excessive modulation frequently uses small or irregular beam segments with large dose calculation uncertainties and accompanies excessive mechanical modulations of MLC positions, gantry rotation, and dose-rates, its use increases both the dose calculation and mechanical uncertainties [11]. These uncertainties lead to discrepancies between plan and delivery, i.e., VMAT delivery accuracy becomes poor [10–12]. In this regard, pre-treatment verification of planned VMAT delivery accuracy is highly recommended for each patient, and therefore pre-treatment, patient-specific quality assurance (QA) for VMAT is routinely performed in clinical settings [10].

The most widely adopted patient-specific QA method is the gamma-index technique suggested by Low et al. [13]. For VMAT, the global gamma-index approach with a gamma criterion of 2%/2 mm and 90% passing rate has been recommended by several previous studies [14, 15]. Although it is convenient and practical, recent studies have taken issue with the clinical irrelevance of gamma passing rates [12, 16, 17]. As an alternative to the gamma-index method, machine log file analysis has been recommended by several studies [17, 18]. By analyzing the differences between the original plan and delivery records from the machine log file, the delivery accuracy of VMAT can be identified. However, independent verification of VMAT delivery accuracy cannot be performed with this method because the machine log file is acquired from the linac control system. Thus, several studies have suggested calculating the modulation degree of VMAT plans for predicting VMAT delivery accuracy [11, 19–22].

As an indicator of the VMAT modulation degree, various modulation indices have been presented in the previous studies [11, 19–23]. Modulation indices can reduce resource usage in clinical settings since they can be calculated during planning, i.e., an actual plan delivery or dosimeter setup for verification of VMAT plans are not required. Masi et al. proposed the modulation complexity score for VMAT (MCS_v_) to evaluate the MLC movement variability and beam aperture shape variability of VMAT plans [20]. Li and Xing presented a modulation index to support station-parameter-optimized radiation therapy (MI_SPORT_), which quantifies MLC positional movements weighted by segmental monitor units (MU) at each control point of VMAT plans [21]. Younge et al. suggested the aperture complexity metric which evaluates modulation degree of VMAT by summing MU-weighted aperture perimeter-to-area ratio [22]. As a modulation index for VMAT, we also proposed textural features calculated from the fluence maps of VMAT plans in a previous study [23]. We demonstrated that two textural features (contrast and variance, with a displacement distance *d* = 1) showed superior performance to MCS_v_ and MI_SPORT_ in assessing the VMAT modulation. Those two textural features were strongly correlated with various measures of VMAT delivery accuracy such as gamma passing rates and the results of machine log file analyses.

Although the textural features calculated from the VMAT fluence maps showed considerable potential to be used as modulation indices, no comprehensive performance test has been performed. A previous study on textural features was performed with a single dosimeter and linac model [23]. Moreover, its analyzed treatment sites were limited, including only head and neck (H&N) and prostate cases. Therefore, in this study, for a comprehensive evaluation of the use of textural features as indicators of VMAT delivery accuracy, we tested their performance by utilizing two types of dosimeters, two types of linac models, and VMAT plans with various treatment sites. To acquire reliable results, a total of 240 VMAT plans were analyzed in this study.

## Methods

### Patient selection and simulation

After institutional review board approval, 200 patients were retrospectively selected for this study. Sixty patients with H&N cancer, 40 patients with prostate cancer, 31 patients with liver cancer, 29 patients with spine tumors, 20 patients with brain tumors, and 20 patients with lung cancer were selected. All patients underwent CT scans using various immobilization techniques at the treatment sites using the Brilliance CT Big Bore™ (Phillips, Amsterdam, The Netherlands).

### Treatment planning

Among the 200 patients analyzed, half were treated using the Trilogy™ system with a Millennium 120™ MLC while the other half were treated using the TrueBeam STx™ with a high-definition (HD) 120™ MLC (Varian Medical Systems, Palo Alto, CA, USA).

For patients treated with the Trilogy, 140 VMAT plans were generated, comprising 40 H&N, 40 prostate primary, 40 prostate boost, 11 liver, and 9 spine plans. The H&N VMAT plans were generated with the simultaneous integrated boost (SIB) technique using a total of 3 planning target volumes (PTVs) with prescription doses of 67.5 Gy, 54 Gy, and 48 Gy in 30 fractions. For H&N VMAT plans, 6 MV photon beams were used, while for the other Trilogy plans, 15 MV photon beams were employed. For the patients with prostate cancer, a primary plan with a prescription dose of 50.4 Gy was delivered to a patient in 28 fractions. The target volumes of the primary plans included prostate and seminal vesicles. After that, a boost plan with a prescription dose of 30.6 Gy was delivered in 17 fractions. The target volumes of the boost plans included only the prostate. The prescription doses for patients with liver cancer and spine tumors were 50 Gy in 20 fractions and 30 Gy in 10 fractions, respectively.

For patients treated with the TrueBeam STx, 100 VMAT plans were generated, comprising 20 H&N, 20 brain, 20 stereotactic ablative radiotherapy (SABR) lung cancer, 20 spine SABR, and 20 liver SABR plans. For H&N and brain VMAT plans, 6 MV photon beams were used. For lung SABR VMAT plans, 6 MV flattening filter free (FFF) photon beams were used while 10 MV FFF photon beams were used for both the spine and liver SABR VMAT plans. The H&N VMAT plans were generated with the SIB technique and the prescription doses of the H&N VMAT plans were the same as those with the Trilogy. The prescription dose used in the brain VMAT plans was 30 Gy in 10 fractions. The prescription doses in the lung, spine, and liver SABR VMAT plans were 60 Gy in 4 fractions, 16 Gy in a single fraction, and 42 Gy in 3 fractions, respectively. Information of VMAT plans analyzed in this study is summarized in Table 1.

**Table 1:** Summary of volumetric modulated arc therapy plan information

Treatment site	*N*	Photon energy	Prescription dose (Gy)	Fraction number
Trilogy				
H&N	40	6 MV	67.5, 54, 48 (for PTV1, PTV2, PTV3, respectively)	30
Prostate (PP)	40	15 MV	50.4	28
Prostate (BP)	40	15 MV	30.6	17
Liver	11	15 MV	50	20
Spine	9	15 MV	30	10
TrueBeam STx				
H&N	20	6 MV	67.5, 54, 48 (for PTV1, PTV2, PTV3, respectively)	30
Brain	20	6 MV	30	10
Lung (SABR)	20	6 MV FFF	60	4
Spine (SABR)	20	10 MV FFF	16	1
Liver (SABR)	20	10 MV FFF	42	3

*H&N* head and neck, *PTV* planning target volume, *PP* primary plan, *BP* boost plan, *SABR* stereotactic ablative radiotherapy, *FFF* flattening filter free

To generate the VMAT plans with both the Trilogy and TrueBeam STx systems, the Eclipse™ system (Varian Medical Systems, Palo Alto, CA, USA) was used. Progressive resolution optimizer 3 (PRO3, version 13.7, Varian Medical Systems, Palo Alto, CA, USA) was used for VMAT optimization and the anisotropic analytic algorithm (AAA, version 13.7, Varian Medical Systems, Palo Alto, CA, USA) was used for dose calculation. When calculating dose distributions of VMAT plans, a dose calculation grid size of 1 mm was always used.

### Texture analysis on the fluence maps of VMAT plans

**Fig. 1:**
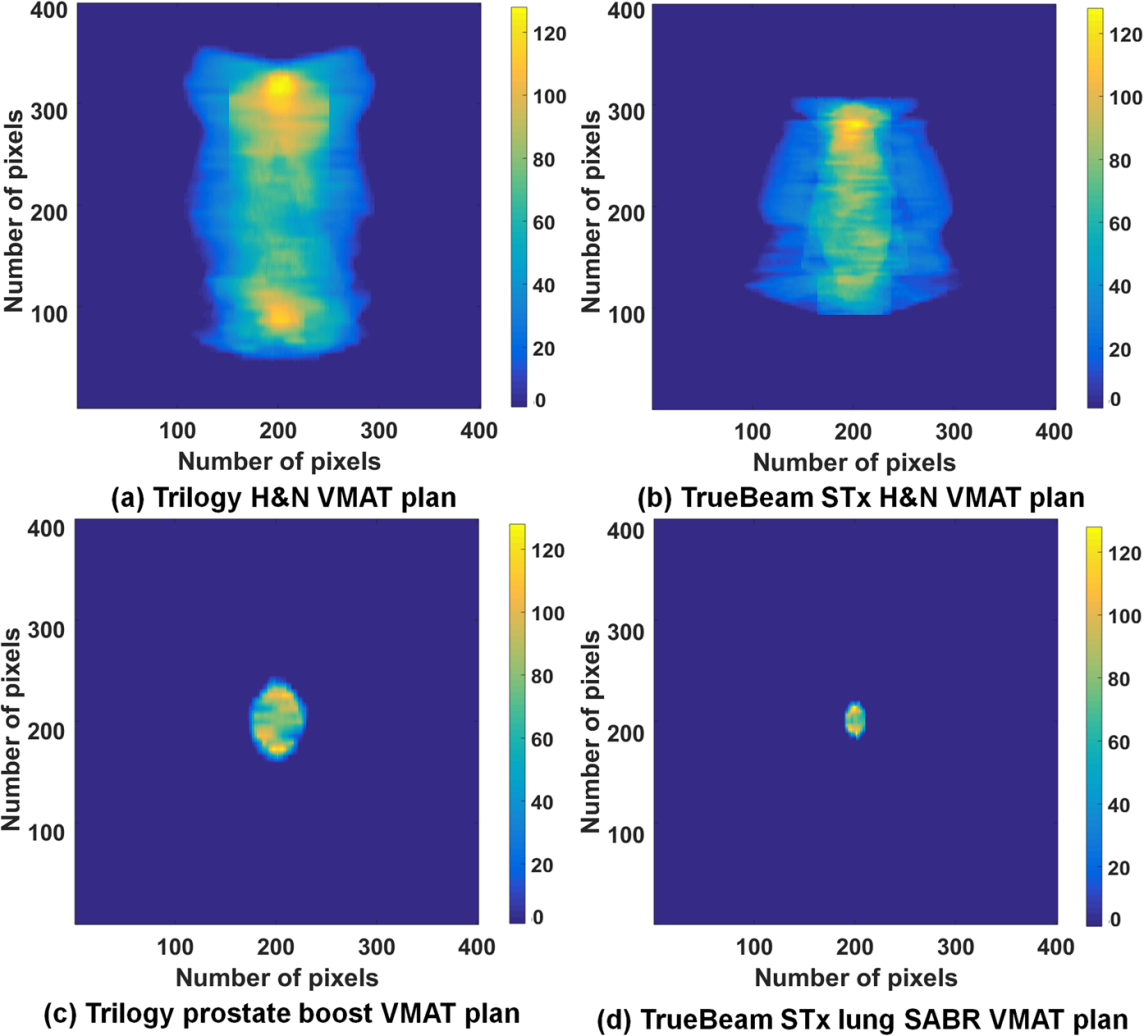
Fluence maps of head and neck (H&N) volumetric modulated arc therapy (VMAT) plans generated with the (**a**) Trilogy and (**b**) TrueBeam STx systems. Fluence maps of a (**c**) prostate boost VMAT plan generated with the Trilogy system and (**d**) that of lung stereotactic ablative radiotherapy (SABR) VMAT plan generated with the TrueBeam STx systems. Fluence maps were generated by the superposition of all fluences at each control point and normalized to pixel values ranging from 0 to 127

**Fig. 2:**
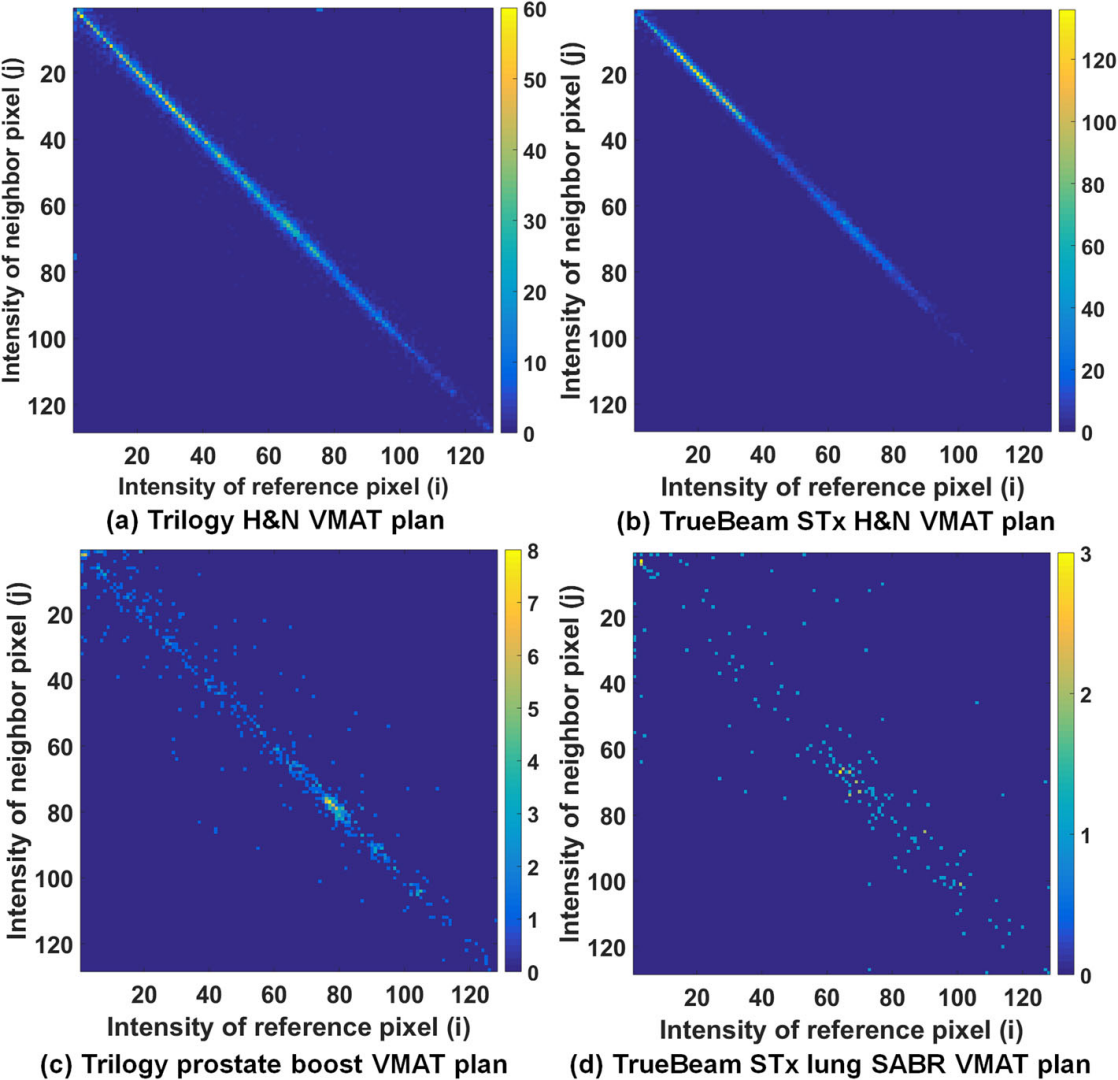
Gray-level co-occurrence matrices (GLCM) of a head and neck (H&M) volumetric modulated arc therapy (VMAT) plan generated with the (**a**) Trilogy and (**b**) the TrueBeam STx systems. **c** GLCM of a prostate boost VMAT plan generated with the Trilogy system. **d** GLCM of a lung stereotactic ablative radiotherapy (SABR) VMAT plan

All VMAT plans in this study were exported from the Eclipse system in DICOM format to generate fluence maps (resolution of 1 mm). With MLC positions and corresponding MUs at each control point from the DICOM formatted files, a fluence map for each VMAT plan was generated by the superposition of each fluence at control points using an in-house program written in MATLAB (version 8.1, Mathworks, Inc., Natick, MA, USA). Then, each fluence map pixel was normalized to a gray level ranging from 0 to 127, so that each map had a maximum gray level of 127. As shown in Fig. 1, examples of fluence maps were generated by integrating all fluences at all control points with the Trilogy and TrueBeam STx. With the normalized fluence maps, a gray-level co-occurrence matrix (GLCM) was generated for each VMAT plan. When generating the GLCM, the particular displacement distances (*d*) which are distances between the reference pixel and neighbor pixels were 1, 5, and 10. For each *d* value, the angles (*θ*) indicating the search directions of the intensity relationship in the fluence maps were 0°, 45°, 90°, and 135° as described in [23]. Therefore, the relationships between pairs of pixels were investigated at the distance of 1, 5, and 10 mm in the horizontal and vertical directions and √2, 5√2, and 10√2 mm for diagonal direction since the resolution of the fluence maps was 1 mm. Examples of generated GLCMs using the Trilogy and TrueBeam STx are shown in Fig. 2. With the GLCM, a total of six textural features for each VMAT plan were calculated as described in [23]. The calculated textural features were angular second moment (ASM), inverse difference moment (IDM), contrast, variance, correlation, and entropy. The ASM is a measure of the homogeneity of a fluence map while the IDM is a measure of its local homogeneity. Contrast is a measure of the local variation in a fluence map and variance is a measure of its inhomogeneity. The correlation measures the linear dependence of the gray levels in a fluence map and entropy measures its randomness. For each value of *d*, the textural features acquired in those four directions were averaged. Because a single textural feature was calculated for each value of *d* and a total of 3 values of *d* (1, 5, and 10) were adopted in this study, three values were calculated for a single type of textural feature for each VMAT plan. For each VMAT plan, a total of six types of textural features (ASM, IDM, contrast, variance, correlation, and entropy) were calculated, for a total of 18 textural features acquired for each VMAT plan. With 240 VMAT plans, 4320 textural features were analyzed in this study.

### Gamma evaluation of VMAT plans

To measure VMAT delivery accuracy, conventional planar gamma evaluation was performed for each VMAT plan. Global gamma analyses with gamma criteria of 2%/2 mm, 2%/1 mm, 1%/2 mm, and 1%/1 mm were performed. When performing gamma evaluation, absolute doses were used and the points with doses less than 10% of the maximum dose were ignored. As suggested by previous studies, a global gamma passing rate of 90% with a gamma criterion of 2%/2 mm is regarded as the clinically acceptable tolerance level here [14, 15]. Two types of dosimeters were chosen for the gamma-index method, the MapCHECK2™ and ArcCHECK™ dosimeters (Sun Nuclear Corporation, Melbourne, FL, USA). The MapCHECK2 was inserted in the MapPHAN™ and installed on the patient couch during planar dose distribution measurements. Reference dose distributions for each type of dosimeter were generated in the Eclipse system with a dose calculation grid size of 1 mm. For an accurate evaluation, linac output was calibrated using the American Association of Physicists in Medicine (AAPM) Task group 51 protocol [24]. In addition, the MapCHECK2 and ArcCHECK dosimeters were calibrated according to manufacturer protocols. After that, planar dose distributions for gamma evaluation were measured. When performing gamma evaluation on the measured dose distributions with the MapCHECK2 and ArcCHECK2 dosimeters compared to the reference dose distributions, the SNC software (Sun Nuclear Corporation, Melbourne, FL, USA) was used.

### Machine log file analysis to examine linac mechanical accuracy during VMAT delivery

To measure VMAT delivery accuracy, machine log files generated by the linac control system during VMAT delivery were acquired and compared to the original VMAT plans. The machine log files were acquired when performing planar dose distribution measurements for gamma evaluation with the MapCHECK2 and ArcCHECK dosimeters, and therefore two machine log files per VMAT plan were obtained. To facilitate comparison of the machine log files and original VMAT plans, the machine log files were reformatted to DICOM-RT files. For each DICOM-RT formatted log file, the differences in the MLC positions, gantry angles, and MUs were calculated at each control point. Since the MLC positional differences and the MU differences between two machine log files acquired during the MapCHECK2 measurements and the ArcCHECK measurements were less than 0.001 mm and 0.01 MU, respectively, the differences at each control point were then averaged. By averaging the two sets of differences with MapCHECK2 and ArcCHECK, we acquired a single set of average values of the MLC positioning errors, gantry angle errors, and MU errors for each VMAT plan.

### Dose-volumetric parameter difference analysis with machine log files

The DICOM-RT-formatted machine log files were imported to the Eclipse system and dose distributions from the machine log files were calculated using the identical CT images and structures from the original VMAT plans. When calculating dose distributions with the machine log files, i.e., when reconstructing dose distributions with machine log files, the same dose calculation grid size of 1 mm as that used for the dose calculation in the original VMAT plan was used. The differences in the clinically-relevant dose-volumetric parameters between the VMAT plans reconstructed with the log files and the original VMAT plans were calculated. Since two machine log files were acquired for each VMAT plan, two sets of differences in the dose-volumetric parameters were acquired and were averaged. A total of 261 clinically relevant dose-volumetric parameters were examined in this study.

### Performance test of each textural features

To test the performance of each textural feature as a predictor of VMAT delivery accuracy, Spearman’s rank correlation coefficients (*r*) and corresponding *p*-values were calculated between the values of textural features and the conventional measures of VMAT delivery accuracy, which were global gamma passing rates, the differences in the mechanical parameters from the machine log files and the differences in the dose-volumetric parameters between the VMAT plans reconstructed with the machine log files and the original VMAT plans. The *r*-values with *p* <  0.05 were regarded as statistically significant in this study. For the correlation analysis with the dose-volumetric parameter differences, 156 and 152 parameters were analyzed for the Trilogy and the TrueBeam STx, respectively, and so the *r*-values with *p* <  0.05 were counted for each textural feature. For the dose-volumetric parameter analysis, we assumed that the textural feature that most frequently showed statistically-significant *r*-values relative to the dose-volumetric parameter differences was the most superior indicator in predicting VMAT delivery accuracy.

## Results

### Calculated values of each textural features

The calculated values of each textural feature for each treatment site are shown in Table 2. For both the Trilogy and TrueBeam STx systems, the H&N VMAT plans always showed the lowest values of ASM, contrast, and variance while the H&N VMAT plans always showed the highest values of IDM, correlation, and entropy among all textural features, regardless of the value of *d*. An opposite tendency occurred for prostate boost plans with the Trilogy system, which showed the highest values of ASM, contrast, and variance and showed the lowest values of IDM, correlation, and entropy. For the TrueBeam STx, lung SABR VMAT plans showed an opposite tendency to the H&N VMAT plans, showing the highest values of ASM and contrast and the lowest values of IDM, correlation, and entropy.

**Table 2:** Textural features calculated from fluence maps of volumetric modulated arc therapy (VMAT) plans with various treatment sites

Textural feature	*d*	Trilogy				
		H&N (*N* = 40)	Prostate (PP) (*N* = 40)	Prostate (BP) (*N* = 40)	Liver (*N* = 11)	Spine (*N* = 9)
ASM (✕10^− 3^)	1	1.06 ± 0.33	1.66 ± 0.31	2.12 ± 0.34	1.34 ± 0.39	1.58 ± 0.32
	5	0.82 ± 0.23	1.94 ± 0.38	2.55 ± 0.54	1.32 ± 0.63	1.61 ± 0.59
	10	0.97 ± 0.37	2.98 ± 0.86	6.37 ± 2.92	1.91 ± 1.46	2.34 ± 1.09
IDM	1	0.30 ± 0.04	0.25 ± 0.02	0.23 ± 0.02	0.28 ± 0.03	0.28 ± 0.04
	5	0.16 ± 0.02	0.11 ± 0.01	0.10 ± 0.01	0.13 ± 0.02	0.13 ± 0.02
	10	0.12 ± 0.01	0.08 ± 0.01	0.07 ± 0.01	0.10 ± 0.01	0.10 ± 0.01
Contrast (✕10^3^)	1	0.11 ± 0.04	0.30 ± 0.08	0.51 ± 0.11	0.20 ± 0.11	0.20 ± 0.07
	5	0.41 ± 0.13	1.30 ± 0.35	1.44 ± 0.32	0.89 ± 0.33	0.78 ± 0.11
	10	0.62 ± 0.19	2.28 ± 0.63	3.03 ± 0.57	1.46 ± 0.54	1.25 ± 0.28
Variance (✕10)	1	3.84 ± 0.50	4.50 ± 0.55	5.04 ± 0.34	4.92 ± 0.37	4.42 ± 0.31
	5	3.98 ± 0.52	5.13 ± 0.59	5.17 ± 0.40	5.09 ± 0.42	4.51 ± 0.35
	10	4.01 ± 0.54	5.14 ± 0.63	5.60 ± 0.54	5.15 ± 0.41	4.64 ± 0.38
Correlation	1	0.93 ± 0.02	0.88 ± 0.03	0.80 ± 0.04	0.92 ± 0.05	0.90 ± 0.03
	5	0.76 ± 0.07	0.54 ± 0.09	0.48 ± 0.08	0.67 ± 0.12	0.62 ± 0.06
	10	0.64 ± 0.10	0.24 ± 0.15	0.11 ± 0.14	0.46 ± 0.21	0.45 ± 0.06
Entropy	1	3.21 ± 0.09	2.91 ± 0.06	2.76 ± 0.07	3.06 ± 0.15	3.00 ± 0.14
	5	3.33 ± 0.08	2.85 ± 0.07	2.68 ± 0.09	3.10 ± 0.23	3.02 ± 0.22
	10	3.31 ± 0.09	2.67 ± 0.11	2.36 ± 0.18	3.00 ± 0.33	2.88 ± 0.30
		TrueBeam STx				
	*d*	Lung SABR (*N* = 20)	Spine SABR (*N* = 20)	Liver SABR (*N* = 20)	Brain (*N* = 20)	H&N (*N* = 20)
ASM (✕10^− 3^)	1	2.38 ± 0.99	1.46 ± 0.34	1.78 ± 0.62	1.51 ± 0.50	1.00 ± 0.29
	5	3.02 ± 2.00	1.60 ± 0.58	1.94 ± 0.89	1.33 ± 0.60	0.63 ± 0.14
	10	5.47 ± 4.24	2.05 ± 0.79	3.12 ± 2.11	1.72 ± 1.13	0.70 ± 0.18
IDM	1	0.22 ± 0.03	0.25 ± 0.02	0.26 ± 0.03	0.30 ± 0.04	0.31 ± 0.03
	5	0.09 ± 0.02	0.11 ± 0.01	0.11 ± 0.02	0.13 ± 0.03	0.17 ± 0.02
	10	0.10 ± 0.16	0.08 ± 0.01	0.08 ± 0.02	0.09 ± 0.02	0.13 ± 0.02
Contrast (✕10^3^)	1	0.43 ± 0.20	0.19 ± 0.04	0.22 ± 0.08	0.18 ± 0.12	0.09 ± 0.02
	5	1.65 ± 0.39	1.04 ± 0.22	1.18 ± 0.39	1.05 ± 0.50	0.36 ± 0.13
	10	2.86 ± 1.14	1.52 ± 0.37	1.97 ± 0.91	1.71 ± 0.84	0.53 ± 0.19
Variance (✕10)	1	4.83 ± 0.32	4.43 ± 0.32	4.58 ± 0.39	4.94 ± 0.32	3.77 ± 0.37
	5	5.02 ± 0.32	4.60 ± 0.32	4.75 ± 0.40	5.15 ± 0.33	3.87 ± 0.39
	10	5.24 ± 1.02	4.62 ± 0.30	4.85 ± 0.42	5.24 ± 0.38	3.90 ± 0.40
Correlation	1	0.82 ± 0.08	0.90 ± 0.03	0.90 ± 0.03	0.92 ± 0.05	0.94 ± 0.02
	5	0.34 ± 0.15	0.52 ± 0.09	0.50 ± 0.12	0.61 ± 0.18	0.77 ± 0.08
	10	0.04 ± 0.19	0.30 ± 0.14	0.23 ± 0.22	0.39 ± 0.26	0.66 ± 0.12
Entropy	1	2.71 ± 0.19	3.01 ± 0.11	2.89 ± 0.19	3.03 ± 0.17	3.25 ± 0.07
	5	2.65 ± 0.25	3.01 ± 0.15	2.88 ± 0.24	3.10 ± 0.24	3.42 ± 0.06
	10	2.39 ± 0.52	2.92 ± 0.17	2.74 ± 0.32	3.04 ± 0.30	3.43 ± 0.06

*d* particular displacement distance, *H&N* head and neck, *PP* primary plan, *BP* boost plan, *ASM* angular second moment, *IDM* inverse difference moment, *SABR* stereotactic ablative radiotherapy

### Global gamma passing rates of VMAT plans

The global gamma passing rates with gamma criteria of 2%/2 mm, 2%/1 mm, 1%/2 mm, and 1%/1 mm using MapCHECK2 and ArcCHECK dosimeters are shown in Table 3. For the Trilogy system, gamma passing rates with the MapCHECK2 dosimeter indicated that the liver VMAT plans had the highest delivery accuracy while the prostate primary VMAT plans had the lowest delivery accuracy. With the ArcCHECK dosimeter, the delivery accuracy of H&N VMAT plans was the highest while that of the prostate boost VMAT plans was the lowest. For the TrueBeam STx system, the MapCHECK2 measurements indicated that the liver SABR VMAT delivery accuracy was the highest while that of the H&N VMAT plans was the lowest. For the ArcCHECK measurements, the liver SABR VMAT delivery accuracy was the lowest while the H&N VMAT delivery accuracy was the highest.

**Table 3:** Global gamma passing rates of volumetric modulated arc therapy (VMAT) plans with various gamma criteria

Treatment site	2%/2 mm		2%/1 mm		1%/2 mm		1%/1 mm	
	MC	AC	MC	AC	MC	AC	MC	AC
Trilogy								
H&N	96.3 ± 2.4	98.5 ± 2.1	90.1 ± 3.9	94.4 ± 3.7	87.8 ± 5.3	94.8 ± 3.9	72.7 ± 6.9	85.1 ± 6.4
Prostate (PP)	95.3 ± 4.0	96.6 ± 2.8	87.6 ± 7.6	86.6 ± 7.4	89.3 ± 4.6	91.9 ± 4.2	72.2 ± 7.9	75.4 ± 8.2
Prostate (BP)	96.8 ± 1.7	96.0 ± 4.1	91.9 ± 3.2	85.6 ± 7.9	92.0 ± 3.2	91.3 ± 5.9	79.1 ± 5.6	74.5 ± 9.8
Liver	97.3 ± 2.6	98.2 ± 1.4	93.9 ± 3.9	92.3 ± 4.2	90.4 ± 4.1	94.5 ± 2.9	80.1 ± 6.4	82.9 ± 5.8
Spine	96.4 ± 3.4	97.3 ± 1.6	91.5 ± 6.6	89.3 ± 6.8	91.0 ± 5.5	92.5 ± 2.5	79.0 ± 8.6	78.4 ± 7.7
TrueBeam STx								
Lung (SABR)	98.6 ± 1.5	99.0 ± 0.6	93.6 ± 2.9	93.2 ± 3.4	93.6 ± 3.8	97.5 ± 0.8	81.2 ± 6.4	89.2 ± 3.7
Spine (SABR)	98.8 ± 1.2	98.9 ± 0.9	95.7 ± 2.4	95.3 ± 2.5	95.4 ± 2.5	97.1 ± 1.6	88.6 ± 4.2	89.2 ± 3.9
Liver (SABR)	98.9 ± 1.3	98.6 ± 1.4	96.0 ± 2.9	93.7 ± 4.8	96.0 ± 2.3	96.5 ± 2.8	87.6 ± 6.5	87.3 ± 6.8
Brain	97.6 ± 2.1	99.5 ± 0.6	95.0 ± 2.4	95.4 ± 5.0	92.5 ± 3.5	98.0 ± 1.6	84.5 ± 3.8	89.4 ± 7.2
H&N	97.5 ± 1.6	99.6 ± 0.8	92.2 ± 3.8	98.3 ± 2.0	89.9 ± 3.1	97.4 ± 3.0	75.6 ± 5.7	93.1 ± 5.6

*MC* MapCHECK2 measurements, *AC* ArcCHECK measurements, *H&N* head and neck, *PP* primary plan, *BP* boost plan, *SABR* stereotactic ablative radiotherapy

### Mechanical errors of VMAT plans

The mechanical errors in VMAT plans from the Trilogy and TrueBeam STx systems are shown in Table 4. For both the Trilogy and TrueBeam STx systems, MLC errors of the H&N VMAT plans were largest. For the Trilogy system, MLC errors of prostate boost plans were smallest while the MLC errors of liver SABR VMAT plans were smallest for the TrueBeam STx system. The gantry angle errors of the prostate primary plans and MU errors of the prostate boost plans were smallest among those from the Trilogy system. For the TrueBeam STx system, the gantry angle errors of the lung SABR VMAT plans and the MU errors of the H&N VMAT plans were smallest among all errors.

**Table 4:** Differences in mechanical parameters between machine log files and original volumetric modulated arc therapy (VMAT) plans

Treatment site	MLC error (mm)	Gantry angle error (°)	Monitor unit error (MU)
Trilogy			
H&N	0.188 ± 0.065	0.054 ± 0.002	0.104 ± 0.072
Prostate (PP)	0.043 ± 0.082	0.053 ± 0.004	0.061 ± 0.021
Prostate (BP)	0.027 ± 0.011	0.054 ± 0.002	0.055 ± 0.011
Liver	0.122 ± 0.098	0.054 ± 0.002	0.076 ± 0.037
Spine	0.073 ± 0.061	0.054 ± 0.001	0.124 ± 0.042
TrueBeam STx			
H&N	0.090 ± 0.012	0.029 ± 0.000	0.017 ± 0.005
Brain	0.040 ± 0.022	0.029 ± 0.002	0.052 ± 0.041
Lung (SABR)	0.011 ± 0.006	0.010 ± 0.001	0.396 ± 0.033
Spine (SABR)	0.024 ± 0.011	0.025 ± 0.003	0.257 ± 0.088
Liver (SABR)	0.029 ± 0.018	0.037 ± 0.025	0.189 ± 0.106

*MLC* Multi-leaf collimator, *H&N* head and neck, *PP* primary plan, *BP* boost plan, *SABR* stereotactic ablative radiotherapy

### Correlations between the values of textural features and global gamma passing rates

The correlations between the values of various textural features and global gamma passing rates of the Trilogy system are shown in Table 5. Only *r*-values with *p* <  0.05 are shown. In general, the variance (*d* = 5 and 10) was correlated with global gamma passing rates with various gamma criteria for the MapCHECK2 measurements, but the correlations were not strong with *r* <  0.4. For the ArcCHECK measurements, the IDM (*d* = 1) and correlation (*d* = 1 and 10) were generally correlated with the global gamma passing rates with various gamma criteria (absolute *r*-values ranging from 0.306 to 0.589).

**Table 5:** Correlations between textural features and global gamma passing rates for the Trilogy system

Textural features	*d*	2%/2 mm		2%/1 mm		1%/2 mm		1%/1 mm	
		*r*	*p*	*r*	*p*	*r*	*p*	*r*	*p*
MapCHECK2 measurements									
ASM	1	–	–	–	–	0.331	< 0.001	0.259	0.002
	5	–	–	–	–	0.313	< 0.001	0.207	0.014
	10	–	–	–	–	0.358	< 0.001	0.261	0.002
IDM	1	–	–	–	–	− 0.198	0.019	–	–
	5	–	–	–	–	−0.336	< 0.001	−0.239	0.004
	10	–	–	–	–	− 0.298	< 0.001	−0.200	0.018
Contrast	1	–	–	–	–	0.322	< 0.001	0.246	0.003
	5	–	–	–	–	0.355	< 0.001	0.268	0.001
	10	–	–	–	–	0.382	< 0.001	0.284	0.001
Variance	1	–	–	0.192	0.023	0.319	< 0.001	0.284	0.001
	5	–	–	0.213	0.012	0.337	< 0.001	0.300	< 0.001
	10	–	–	0.250	0.003	0.349	< 0.001	0.342	< 0.001
Correlation	1	–	–	–	–	−0.229	0.006	–	–
	5	–	–	–	–	−0.315	< 0.001	− 0.201	0.017
	10	–	–	–	–	−0.348	< 0.001	−0.187	0.027
Entropy	1	–	–	–	–	−0.344	< 0.001	−0.272	0.001
	5	–	–	–	–	−0.360	< 0.001	−0.269	0.001
	10	–	–	–	–	−0.372	< 0.001	−0.281	0.001
ArcCHECK measurements									
ASM	1	0.337	< 0.001	0.471	< 0.001	0.235	0.005	0.402	< 0.001
	5	0.383	< 0.001	0.551	< 0.001	0.268	0.001	0.483	< 0.001
	10	0.393	< 0.001	0.560	< 0.001	0.273	0.001	0.489	< 0.001
IDM	1	−0.422	< 0.001	−0.589	< 0.001	−0.350	< 0.001	−0.561	< 0.001
	5	−0.421	< 0.001	− 0.540	< 0.001	−0.321	< 0.001	−0.487	< 0.001
	10	−0.429	< 0.001	−0.500	< 0.001	−0.357	< 0.001	−0.472	< 0.001
Contrast	1	0.432	< 0.001	0.552	< 0.001	0.333	< 0.001	0.508	< 0.001
	5	0.375	< 0.001	0.486	< 0.001	0.296	< 0.001	0.452	< 0.001
	10	0.393	< 0.001	0.519	< 0.001	0.309	< 0.001	0.470	< 0.001
Variance	1	0.281	0.001	0.344	< 0.001	0.228	0.007	0.327	< 0.001
	5	0.269	0.001	0.326	< 0.001	0.210	0.013	0.310	< 0.001
	10	0.301	< 0.001	0.330	< 0.001	0.246	0.003	0.321	< 0.001
Correlation	1	−0.457	< 0.001	−0.582	< 0.001	− 0.358	< 0.001	− 0.540	< 0.001
	5	− 0.415	< 0.001	− 0.543	< 0.001	−0.328	< 0.001	−0.502	< 0.001
	10	−0.414	< 0.001	−0.582	< 0.001	−0.306	< 0.001	−0.517	< 0.001
Entropy	1	−0.382	< 0.001	−0.525	< 0.001	−0.267	0.001	−0.460	< 0.001
	5	−0.385	< 0.001	−0.543	< 0.001	−0.266	0.001	−0.476	< 0.001
	10	−0.384	< 0.001	−0.544	< 0.001	−0.261	0.002	−0.475	< 0.001

*d* particular displacement distance, *r* Spearman’s rank correlation coefficient, *ASM* angular second moment, *IDM* inverse difference moment

The statistically significant correlations (with *p* <  0.05) between the values of various textural features and global gamma passing rates of the TrueBeam STx system are shown in Table 6. For the MapCHECK2 measurements, ASM (*d* = 5 and 10) and IDM (*d* = 5 and 10) generally showed good correlations with global gamma passing rates (absolute *r*-values ranging from 0.347 to 0.546). For the ArcCHECK measurements, IDM (*d* = 1), ASM (*d* = 10), and entropy (*d* = 5 and 10) were generally correlated strongly to gamma passing rates (absolute *r*-values ranging from 0.238 to 0.614).

**Table 6:** Correlations between textural features and global gamma passing rates of the TrueBeam STx system

Textural features	*d*	2%/2 mm		2%/1 mm		1%/2 mm		1%/1 mm	
		*r*	*p*	*r*	*p*	*r*	*p*	*r*	*p*
MapCHECK2 measurements									
ASM	1	0.274	0.006	–	–	0.406	< 0.001	0.321	0.001
	5	0.347	< 0.001	–	–	0.531	< 0.001	0.407	< 0.001
	10	0.351	< 0.001	–	–	0.519	< 0.001	0.385	< 0.001
IDM	1	−0.333	0.001	–	–	−0.473	< 0.001	− 0.300	0.002
	5	−0.395	< 0.001	–	–	−0.538	< 0.001	−0.400	< 0.001
	10	−0.381	< 0.001	–	–	−0.546	< 0.001	−0.398	< 0.001
Contrast	1	0.337	0.001	–	–	0.432	< 0.001	0.277	0.005
	5	0.374	< 0.001	–	–	0.439	< 0.001	0.334	0.001
	10	0.311	0.002	–	–	0.441	< 0.001	0.336	0.001
Variance	1	0.213	0.033	0.202	0.044	0.262	0.008	0.339	0.001
	5	0.223	0.026	0.202	0.044	0.263	0.008	0.331	0.001
	10	–	–	–	–	0.234	0.019	0.247	0.013
Correlation	1	−0.254	0.011	–	–	−0.349	< 0.001	–	–
	5	−0.318	0.001	–	–	− 0.409	< 0.001	− 0.251	0.012
	10	−0.313	0.002	–	–	−0.468	< 0.001	−0.337	0.001
Entropy	1	−0.335	0.001	–	–	−0.478	< 0.001	− 0.350	< 0.001
	5	−0.343	< 0.001	–	–	−0.506	< 0.001	−0.378	< 0.001
	10	−0.352	< 0.001	–	–	−0.510	< 0.001	−0.377	< 0.001
ArcCHECK measurements									
ASM	1	0.371	0.001	0.467	< 0.001	–	–	0.256	0.010
	5	0.418	< 0.001	0.580	< 0.001	0.229	0.022	0.369	< 0.001
	10	0.448	< 0.001	0.614	< 0.001	0.259	0.009	0.398	< 0.001
IDM	1	−0.475	< 0.001	−0.562	< 0.001	−0.300	0.002	−0.380	< 0.001
	5	−0.415	< 0.001	−0.537	< 0.001	−0.260	0.009	−0.352	< 0.001
	10	−0.398	< 0.001	−0.526	< 0.001	−0.259	0.009	−0.367	< 0.001
Contrast	1	0.397	< 0.001	0.532	< 0.001	0.209	0.037	0.311	0.002
	5	0.370	< 0.001	0.519	< 0.001	–	–	0.296	0.003
	10	0.375	< 0.001	0.519	< 0.001	–	–	0.322	0.001
Variance	1	–	–	0.252	0.011	–	–	–	–
	5	–	–	0.257	0.010	–	–	–	–
	10	–	–	0.293	0.003	–	–	–	–
Correlation	1	−0.432	< 0.001	−0.521	< 0.001	−0.299	0.002	−0.349	< 0.001
	5	−0.422	< 0.001	−0.530	< 0.001	−0.247	0.013	−0.319	0.001
	10	−0.463	< 0.001	−0.567	< 0.001	−0.262	0.009	−0.366	< 0.001
Entropy	1	−0.418	< 0.001	−0.585	< 0.001	−0.218	0.029	−0.362	< 0.001
	5	−0.441	< 0.001	−0.602	< 0.001	−0.238	0.017	−0.381	< 0.001
	10	−0.447	< 0.001	−0.610	< 0.001	− 0.250	0.012	−0.390	< 0.001

*d* particular displacement distance, *r* Spearman’s rank correlation coefficient, *ASM* angular second moment, *IDM* inverse difference moment

### Correlations between the values of textural features and mechanical errors during plan delivery

The statistically significant correlations (with *p* <  0.05) between the textural feature values and mechanical errors during VMAT plan delivery are shown in Table 7. For the Trilogy system, ASM (*d* = 5 and 10), correlation (*d* = 1 and 10), and entropy (*d* = 1, 5, and 10) showed strong correlations with the MLC errors, with absolute *r* > 0.8. The contrast (*d* = 5) and variance (*d* = 1, 5, and 10) showed absolute *r* > 0.7 for the MU delivery errors. For the TrueBeam STx system, ASM (*d* = 5 and 10) and entropy (*d* = 1, 5, and 10) were strongly correlated to the MLC errors with absolute *r* > 0.85. For the gantry angle errors, IDM (*d* = 1) were also strongly correlated with absolute *r* > 0.5. The IDM (*d* = 1), contrast (*d* = 1), correlation (*d* = 1, 5, and 10), and entropy (*d* = 5 and 10) were likewise strongly correlated to the MU delivery errors with absolute *r* > 0.6.

**Table 7:** Correlations between textural features and mechanical errors

Textural feature	*d*	MLC error		Gantry angle error		MU error	
		*r*	*p*	*r*	*p*	*r*	*p*
Trilogy							
ASM	1	− 0.767	< 0.001	–	–	−0.382	< 0.001
	5	−0.860	< 0.001	–	–	−0.417	< 0.001
	10	−0.843	< 0.001	–	–	−0.482	< 0.001
IDM	1	0.788	< 0.001	–	–	0.391	< 0.001
	5	0.679	< 0.001	–	–	0.657	< 0.001
	10	0.599	< 0.001	–	–	0.640	< 0.001
Contrast	1	−0.759	< 0.001	–	–	−0.588	< 0.001
	5	−0.617	< 0.001	–	–	−0.718	< 0.001
	10	−0.688	< 0.001	–	–	−0.688	< 0.001
Variance	1	−0.441	< 0.001	−0.199	0.018	−0.718	< 0.001
	5	−0.401	< 0.001	−0.215	0.011	−0.746	< 0.001
	10	−0.429	< 0.001	−0.200	0.018	−0.722	< 0.001
Correlation	1	0.807	< 0.001	–	–	0.394	< 0.001
	5	0.717	< 0.001	–	–	0.542	< 0.001
	10	0.802	< 0.001	–	–	0.522	< 0.001
Entropy	1	0.811	< 0.001	–	–	0.520	< 0.001
	5	0.836	< 0.001	–	–	0.516	< 0.001
	10	0.830	< 0.001	–	–	0.529	< 0.001
TrueBeam STx							
ASM	1	−0.780	< 0.001	−0.313	< 0.001	−0.440	< 0.001
	5	− 0.858	< 0.001	−0.404	< 0.001	−0.572	< 0.001
	10	−0.859	< 0.001	−0.411	< 0.001	−0.589	< 0.001
IDM	1	0.747	< 0.001	0.507	< 0.001	0.673	< 0.001
	5	0.805	< 0.001	0.399	< 0.001	0.589	< 0.001
	10	0.744	< 0.001	0.280	0.005	0.505	< 0.001
Contrast	1	−0.829	< 0.001	−0.490	< 0.001	− 0.649	< 0.001
	5	−0.843	< 0.001	−0.403	< 0.001	−0.579	< 0.001
	10	−0.819	< 0.001	−0.353	< 0.001	−0.545	< 0.001
Variance	1	−0.541	< 0.001	–	–	−0.209	0.037
	5	−0.536	< 0.001	–	–	−0.211	0.035
	10	−0.607	< 0.001	−0.214	0.033	−0.292	0.003
Correlation	1	0.695	< 0.001	0.483	< 0.001	0.617	< 0.001
	5	0.802	< 0.001	0.438	< 0.001	0.627	< 0.001
	10	0.819	< 0.001	0.411	< 0.001	0.608	< 0.001
Entropy	1	0.873	< 0.001	0.404	< 0.001	0.579	< 0.001
	5	0.885	< 0.001	0.437	< 0.001	0.616	< 0.001
	10	0.887	< 0.001	0.440	< 0.001	0.628	< 0.001

*d* particular displacement distance, *MLC* multi-leaf collimator, *MU* monitor unit, *r* Spearman’s rank correlation coefficient, *ASM* angular second moment, *IDM* inverse difference moment

### Correlations between the values of textural features and dose-volumetric parameter differences

**Fig. 3:**
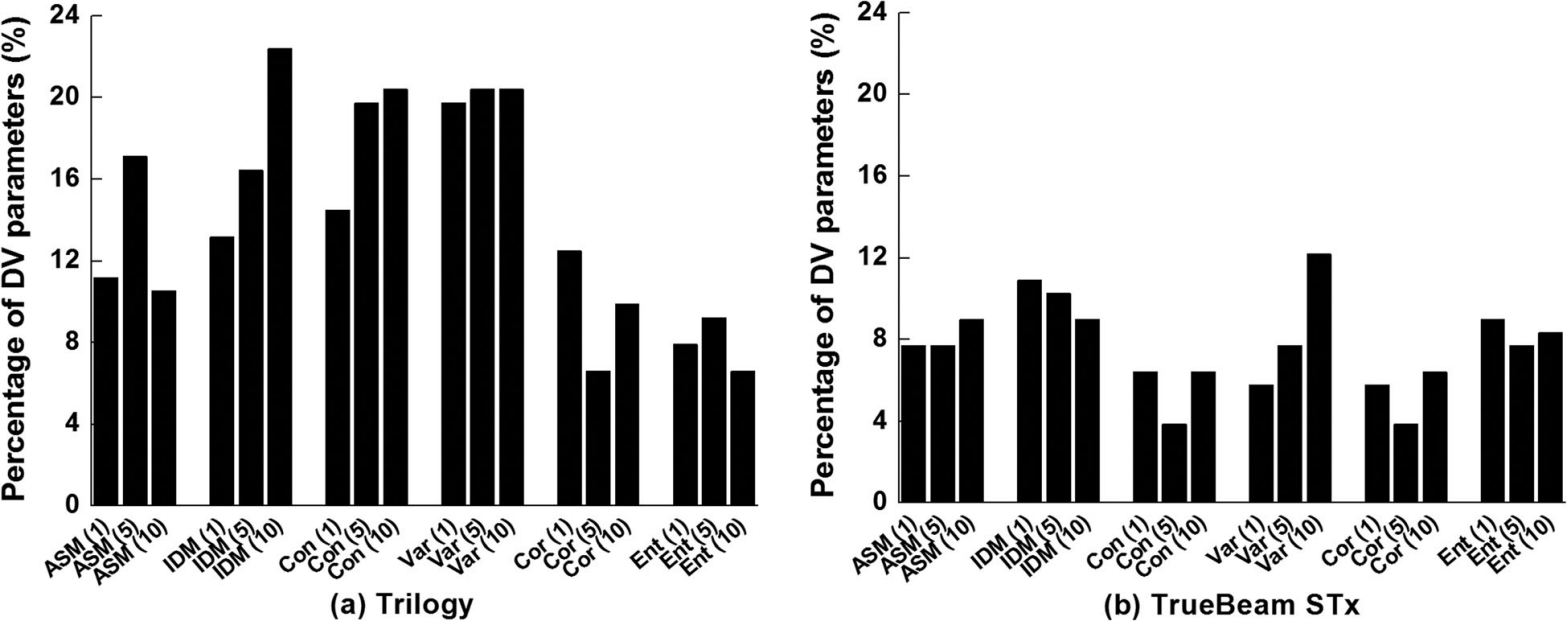
Percentages of dose-volumetric parameters for that Spearman’s rank correlation coefficient (*r*) with *p*-value of less than 0.05 between the textural feature values and dose-volumetric parameter differences are shown for each textural feature for (**a**) the Trilogy and (**b**) the TrueBeam STx. The six texture features which had angular second moment (ASM), inverse difference moment (IDM), contrast, variance, correlation, and entropy were used. The dose-volumetric parameter differences were the differences in the dose-volumetric parameters between original volumetric modulated arc therapy (VMAT) plans and the VMAT plans reconstructed with the machine log files recorded in the linac control system during plan delivery. A total of 156 and 152 dosevolumetric parameters were examined for the Trilogy and the TrueBeam STx, respectively

The numbers of statistically significant *r*-values of each textural feature to the differences in the dose-volumetric parameters between original VMAT plans and the VMAT plans reconstructed with the machine log files are shown in Fig. 3. For the Trilogy system, the IDM (*d* = 10), contrast (*d* = 5 and 10), and variance (*d* = 5 and 10) showed more than 30 statistically-significant *r*-values. For the TrueBeam STx system, the variance (*d* = 10) and IDM (*d* = 1 and 5) demonstrated more than 15 statistically significant *r*-values.

## Discussion

In this study, the performance of various textural features as predictors of VMAT delivery accuracy were comprehensively tested. To review the mechanical parameter differences, the MLC errors of the H&N VMAT plans were highest while those of the prostate VMAT plans were lowest for the Trilogy system, which is consistent with previous studies [9, 11, 15, 23]. In general, the modulation degree of H&N VMAT plans using SIB is high owing to the concave shape of the target volume, multiple target volumes with multiple prescription doses requiring steep dose gradients between the target volumes, and OARs proximal to or overlapped with the target volumes [20, 21]. Compared to the H&N VMAT plans, the modulation degree of prostate VMAT plans has been shown to be relatively low [11, 20, 21]. Therefore, large MLC errors owing to the complicated mechanical movements of MLCs occurred in the H&N VMAT plans while small MLC errors were observed for prostate VMAT plans in this study. For the TrueBeam STx system, the MLC errors of the H&N VMAT plans were largest and those of lung SABR were smallest. This is also consistent with previous studies [9, 25]. For the lung SABR, the target volume is small and generally no OAR proximal to the target volume exists [25]. Consequently, strong correlations generally > 0.7 were observed between the MLC errors and the values of textural features similar to the results of the previous study [23]. The ASM (*d* = 5 and 10) and entropy (*d* = 1, 5 and 10) had absolute *r* > 0.8 (with *p* <  0.001). The correlations of the textural features to the gantry angle errors and MU delivery errors were relatively low compared to the correlations to the MLC errors. The MLC errors dominated the effect on VMAT delivery accuracy while the effects of gantry angle errors and MU delivery errors were minimal in the previous study [26]. Therefore, these kinds of errors are not necessarily considered significant for the performance of textural features in predicting VMAT delivery accuracy.

To review gamma passing rates, no consistent results were observed between the MapCHECK2 and ArcCHECK measurements. Gamma passing rates with the MapCHECK2 measurements for the Trilogy system indicated that primary prostate VMAT plans were modulated highest except for gamma passing rates with 1%/2 mm, contradicts previous studies as well as the mechanical errors in this study [11, 20, 21]. However, gamma passing rates with 1%/2 mm for the Trilogy system and gamma passing rates with every gamma criterion for the TrueBeam STx of the MapCHECK2 measurement indicated that the H&N VMAT plans were modulated highest, which is consistent with previous studies [11, 20, 21]. For the ArcCHECK measurements, gamma passing rates contradicted previous studies and those of the mechanical errors by machine log files for both the Trilogy and TrueBeam STx systems [11, 20, 21]. Therefore, the gamma passing rates in this study were not reliable except for those with 1%/2 mm of the MapCHECK2 measurements. All VMAT plans analyzed in this study were clinically acceptable with much higher global gamma passing rates than 90% with 2%/2 mm, which is the recommended tolerance level for patient-specific QA for VMAT from Heilemann et al. and Fredh et al. [14, 15]. Therefore, the gamma passing rate fluctuations were small and the factors lowering the gamma passing rates might be due to dosimeter setup errors or dosimeter uncertainties, or the spatial resolution of dosimeter, rather than due to the modulation degrees of VMAT plans. In addition, previous studies demonstrated that the gamma passing rate is clinically irrelevant, although it is suitable for detecting IMRT or VMAT plans with significant errors possible to cause medical accidents [16]. A fine distinction between VMAT plans with different delivery accuracy seems difficult with 2D global gamma passing rates. In this respect, gamma passing rates in this study except those with 1%/2 mm were not reliable for quantifying the modulation degrees of VMAT plans. To examine the correlations of the textural features to the gamma passing rates with 1%/2 mm, the ASM (*d* = 10) and entropy (*d* = 5 and 10) showed absolute *r* > 0.35 (with *p* <  0.001) for the Trilogy system and absolute *r* > 0.5 (with *p* <  0.001) for the TrueBeam STx system.

The ASM (*d* = 10) and entropy (*d* = 5 and 10) were correlated to both the MLC errors and the gamma passing rates. However, these textural features did not always perform well in the dose-volumetric parameter differences between the original VMAT plans and those reconstructed with the machine log files. The ASM (*d* = 10) performed relatively poorly in the results of dose-volumetric parameter differences for the Trilogy system, but performed fourth-best for the TrueBeam STx system. Therefore, comprehensively reviewing every measure of VMAT delivery accuracy tested in this study, the ASM (*d* = 10) generally showed good performance as a predictor of VMAT delivery accuracy. Because there is no golden reference methodology to correctly predict VMAT delivery accuracy, even an ideal indicator cannot always show strong correlations to every conventional measure of VMAT delivery accuracy. Although the planar gamma-index method has been widely adopted in clinical settings since being suggested by Low et al. [13], its limitations have been raised in several studies, and include the clinical irrelevance of gamma passing rates, the high dependency of gamma passing rates on dosimeter type, no clear gamma criterion with tolerance levels, a lack of information of 2D dose distributions, and dosimeter setup error dependency [11, 16, 27–32]. Machine log file analysis also is limited by its dependency on the linac control system. This might imply that no single textural feature can always demonstrate strong correlation to every measure of VMAT delivery accuracy. Although we cannot claim that the ASM (*d* = 10) can replace the conventional methodologies to predict VMAT delivery accuracy, such as gamma evaluation and machine log file analysis, at least it can support to detect highly-modulated VMAT plans during planning, which can help to reduce resource usage in clinical settings. Furthermore, the time required for calculating a single textural feature and then evaluating VMAT delivery accuracy was less than 0.1 s in this study, which was fast enough to be executed in optimization process, therefore, the calculation of the textural features could be implanted in the optimization process of VMAT planning to guarantee VMAT delivery accuracy in the future.

In this study, the textural feature showing generally best performance was the ASM (*d* = 10) while a previous study concluded optimal features were the contrast (*d* = 1) and variance (*d* = 1) [23]. By utilizing various linac systems, dosimeters, and VMAT plans with various treatment sites, we acquired different results from those of the previous study which suggested the textural features as a predictor of VMAT delivery accuracy for the first time. The contrast (*d* = 1) still performed well, but the performance of the ASM (*d* = 10) was slightly better than that of contrast (*d* = 1) in general. The variance (*d* = 1) performed well in the gamma passing rates with MapCHECK2 dosimeters and the dose-volumetric parameter differences with the Trilogy system, but it did not always correlate best with every VMAT delivery accuracy. Therefore, the results of this study are not entirely contradictory to those of the previous study. In this study, we were able to demonstrate that the performance of the ASM (*d* = 10) was better than that of the variance (*d* = 1) and contrast (*d* = 1) in more comprehensive situations and when we increased the sample size. Since the ASM is a measure of homogeneity of a fluence map, VMAT plans with homogeneous fluence maps could be delivered accurately as intended (higher values of ASM for the VMAT plans with higher delivery accuracy). This was reasonable because homogeneous fluences are probable to be delivered with large and regular beam segments which reduce dose calculation uncertainty. In addition, the homogeneity of the fluence map was strongly correlated to the mechanical errors as shown in the results. Therefore, it seems that both the dose calculation and mechanical uncertainties of the VMAT plans with homogeneous fluence maps were small. On the other hand, the ASM (*d* = 10) showed better performance than the ASM (*d* = 1) and ASM (*d* = 5). Since the largest MLC leaf width was 10 mm in this study (leaf widths of the Millennium 120 MLC = 5 mm and 10 mm, and those of the HD 120 MLC = 2.5 mm and 5 mm), the largest height of the beamlet was 10 mm in this study. In this respect, the investigation of the intensity relationships in the fluence maps at the distance of 10 mm could be more effective than those with 1 mm and 5 mm. This could be a reason of better performance of the ASM (*d* = 10) than ASM (*d* = 1) and ASM (*d* = 5).

The limitation of the present study is that no tolerance levels or action levels of the ASM (*d* = 10) were provided. Since all VMAT plans analyzed in this study were clinically acceptable, we could not determine the tolerance level in this study. At least, the values of ASM (*d* = 10) in this study were always higher than 0.4 ✕ 10^− 3^, therefore, clinically acceptable VMAT plans should show values of ASM (*d* = 10) higher than 0.4 ✕ 10^− 3^. By utilizing VMAT plans which are not clinically acceptable due to excessive modulations, the tolerance level of the ASM (*d* = 10) will be determined in the future. Another limitation of this study is that the results are only valid for Varian linacs. Further investigation utilizing various types of linacs will be performed in the future.

## Conclusions

In this study, we comprehensively tested the performance of textural features as indicators of VMAT delivery accuracy by correlation analysis. In general, the ASM (*d* = 10) showed better performance than others in predicting VMAT delivery accuracy. The ASM (*d* = 10) could be used as a support tool to evaluate VMAT deliver accuracy at the planning level. This can be advantageous by saving resources in clinical settings because it can be simply calculated during planning.

## Data Availability

Data sharing is not applicable to this article, as no datasets were generated or analyzed during the current study.

## Abbreviations

AAA: Anisotropic analytic algorithm
ASM: Angular second moment
*d*: Particular displacement distance
FFF: Flattening filter free
GLCM: Gray-level co-occurrence matrix
H&N: Head and neck
HD: High-definition
IDM: Inverse difference moment
MLC: Multi-leaf collimator
MU: Monitor unit
OAR: Organs at risk
PRO: Progressive resolution optimizer
PTV: Planning target volume
QA: Quality assurance
*r*: Spearman’s rank correlation coefficient
SABR: Stereotactic ablative radiotherapy
SIB: Simultaneous integrated boost
VMAT: Volumetric modulated arc therapy
